# Aberrant phenotype of circulating antigen presenting cells in giant cell arteritis and polymyalgia rheumatica

**DOI:** 10.3389/fimmu.2023.1201575

**Published:** 2023-08-02

**Authors:** Rosanne D. Reitsema, Bernd-Cornèl Hesselink, Wayel H. Abdulahad, Kornelis S. M. van der Geest, Elisabeth Brouwer, Peter Heeringa, Yannick van Sleen

**Affiliations:** ^1^Department of Rheumatology and Clinical Immunology, University of Groningen, University Medical Center Groningen, Groningen, Netherlands; ^2^School of Medical Sciences, Faculty of Medicine and Health, Örebro University, Örebro, Sweden; ^3^Department of Pathology and Medical Biology, University of Groningen, University Medical Center Groningen, Groningen, Netherlands

**Keywords:** giant cell arteritis, polymyalgia rheumatica, monocytes, dendritic cells, vasculitis

## Abstract

**Background:**

Giant Cell Arteritis (GCA) and Polymyalgia Rheumatica (PMR) are overlapping inflammatory diseases. Antigen-presenting cells (APCs), including monocytes and dendritic cells (DCs), are main contributors to the immunopathology of GCA and PMR. However, little is known about APC phenotypes in the peripheral blood at the time of GCA/PMR diagnosis.

**Methods:**

APCs among peripheral blood mononuclear cells (PBMCs) of treatment-naive GCA and PMR patients were compared to those in age- and sex-matched healthy controls (HCs) using flow cytometry (n=15 in each group). We identified three monocyte subsets, and three DC subsets: plasmacytoid DCs (pDCs), CD141+ conventional DCs (cDC1) and CD1c+ conventional DCs (cDC2). Each of these subsets was analyzed for expression of pattern recognition receptors (TLR2, TLR4), immune checkpoints (CD86, PDL1, CD40) and activation markers (HLA-DR, CD11c).

**Results:**

t-SNE plots revealed a differential clustering of APCs between GCA/PMR and HCs. Further analyses showed shifts in monocyte subsets and a lower proportion of the small population of cDC1 cells in GCA/PMR, whereas cDC2 proportions correlated negatively with CRP (r=-0.52). Classical monocytes of GCA/PMR patients show reduced expression of TLR2, HLA-DR, CD11c, which was in contrast to non-classical monocytes that showed higher marker expression. Additionally, single cell RNA sequencing in GCA patients identified a number of differentially expressed genes related to inflammation and metabolism in APCs.

**Conclusion:**

Circulating non-classical monocytes display an activated phenotype in GCA/PMR patients at diagnosis, whereas classical monocytes show reduced expression of activation markers. Whether these findings reflect APC migration patterns or the effects of long-term inflammation remains to be investigated.

## 1 Introduction

Giant cell arteritis (GCA) and polymyalgia rheumatica (PMR) are overlapping, inflammatory diseases affecting people older than 50 years of age. GCA is characterized by inflammation of larger-sized arteries which can lead to symptoms such as headaches, jaw or limb claudication, and vision loss. Approximately 50% of GCA patients have overlapping PMR, which causes pain and stiffness in shoulders and hips due to inflammation of bursae and tendon sheaths. GCA and PMR have been considered to be both part of the same clinical syndrome, coined GPSD (GCA-PMR Spectrum Disease), with PMR being one of the manifestations with a more systemic inflammatory response, but without occurrence of vasculitis (1, 2).

GCA is hypothesized to develop in the adventitia of the vessel wall, where dendritic cells (DCs) become activated via binding of an unknown ligand to their pattern recognition receptors, e.g. toll-like receptors (TLRs) (3). These receptors are essential for sensing pathogen associated molecular patterns (PAMPs), expressed by bacteria and viruses, but also damage associated molecular patterns (DAMPs), which for instance are released by necrotic cells (4). In GCA, DCs may be more prone to activation due to a defect in programmed death ligand 1 (PDL1) expression (5, 6). PDL1 is one of several immune checkpoints that has been implicated in GCA (7). Immune checkpoint ligand and receptor interactions between lymphocytes and antigen presenting cells (APCs) are crucial for regulating immune responses (8). Signals through co-inhibitory immune checkpoints such as via the PD1/PDL1 pathway dampen the immune response whereas stimulatory immune checkpoint interactions such as via CD28/CD80-CD86 and CD40L/CD40 induce immune activation.

In GCA, activation of DCs lacking in PDL1 supposedly leads to chemokine production and recruitment of CD4+ T cells and monocytes to the arterial wall (3). In circulation, three subsets of monocytes can be identified based on CD14/CD16 expression: classical monocytes, the most common subset, intermediate monocytes, which typically express high levels of activation markers, and non-classical monocytes, whose exact function remains debated (9). Recent studies showed that CD4+ T cells and macrophages are present in tissues of inflamed bursae and tendon sheath of PMR patients as well (10, 11). The infiltrated cells in inflamed tissues of GCA and PMR patients in turn produce chemokines and cytokines, such as IL-6, that may further fuel the infiltration and inflammation in the vessel wall. These cytokines also contribute to systemic inflammation, as evidenced by high levels of C-reactive protein (CRP), in patients with GCA and PMR.

The exact role of circulating DC subsets in inflammatory diseases such as GCA and PMR remains unclear. Frequencies of conventional DCs (cDCs) are 5-10 times higher in GCA arteries than in healthy arteries, suggesting massive recruitment of cDCs to the vessel wall during active disease (12). These cells express CCR7 and are thought to be retained in the vessel wall due to high local production of the CCR7 ligands CCL19 and CCL21. In PMR, it is likely that cDCs migrate to the inflamed synovium in these patients. Different functions and phenotypes have been ascribed to the CD11c+ cDCs and the CD303+ pDCs (13). DCs detect PAMPs and DAMPs through pattern recognition receptors, leading to activation and maturation, including the upregulation of CD83, CD86 and MHC-II molecules (e.g. HLA-DR) (5, 12, 13).The function of circulating CD141+ cDC1 is still debated (14, 15), but they are thought to play a role in mediating the efficient recognition of viral and intracellular antigens and subsequently the production of type III interferon. In blood, CD1c+ cDC2 are much more common. They are excellent cross-presenting cells, as can be appreciated by their high HLA-DR expression, and have the capacity to produce a wide range of pro-inflammatory/T-cell skewing cytokines. It has been postulated that the cDC1 subset is responsible for CD8+ T cell activation, whereas the cDC2 subset preferably interacts with CD4+ T cells. In contrast, pDCs have lower antigen presentation capabilities, but produce large amounts of type I IFN and pro-inflammatory cytokines in response to pathogens (16). Compared to cDCs, pDCs have a drastically impaired capacity to migrate to the inflammatory site in response to inflammatory chemotactic chemokines (17).

Although APCs, including monocytes and DCs, seem to be involved in the local inflammatory response in GCA and PMR, their phenotype in blood is largely unknown. In this exploratory study, we investigated whether circulating APCs have an aberrant phenotype and numerical composition at the time of GCA/PMR diagnosis. To this end, expression of TLRs, activation markers and immune checkpoints on subsets of monocytes and DCs was assessed. The relations between the phenotype of APCs and clinical features were examined as well.

## 2 Materials and methods

### 2.1 Study population

Patients with active GCA and PMR (n=15 each) were enrolled in this study at the time of their diagnosis and before start of treatment. Diagnosis of GCA and PMR was based on clinical signs and symptoms or positive proof by imaging with [^18^F]fluorodeoxyglucose-PET scan and/or ultrasound (**Supplementary Table 1**). GCA diagnoses were further based on positive temporal artery biopsies. Healthy controls (HCs, n=15) were aged- and sex-matched, had no morbidities and received no immunosuppressive medication. All patients and HCs were included in the flow cytometry experiments. TLR4 expression was assessed in n=13 patients and controls. In addition, absolute immune cell counts were determined in 15 HCs, 14 GCA and 13 PMR patients by the XN-9000 (Sysmex, Kobe, Japan), based on size and granularity (diff).

All patients and controls were seen by a clinician before study inclusion. The study was executed in accordance with the declaration of Helsinki and all participants gave their written informed consent. The local medical ethical committee approved of this study (METc2010/222).

### 2.2 Flow cytometry staining

Flow cytometry experiments were executed on cryopreserved peripheral blood mononuclear cells (PBMCs). PBMCS were thawed in RPMI + 10% FCS before staining with fluorescently labelled monoclonal antibodies for 15 minutes (**Supplementary Table 2**). Cells were subsequently fixed with FACS lysing solution for 10 minutes. Cells were washed twice in PBS + BSA before analysis with the BD FACSymphony flow cytometer. Before measuring, cytometer setup and tracking beads were used to normalize between measurements at subsequent dates. Initial compensations were generated using compensation beads and further optimized using the fluorescence minus one (FMO) controls when necessary. The setting of gates was based on FMO controls and biological controls (**Supplementary Figure 1**).

### 2.3 Single-cell RNA sequencing

Single-cell RNA sequencing (scRNAseq) was performed on cryopreserved PBMCs by Single Cell Discoveries (https://www.scdiscoveries.com/, Utrecht, the Netherlands) as described before (18). We have previously studied the transcriptome of T cells in GCA, and of CD8+ T cells specifically. For this study we used the dataset generated to analyze clusters containing CD16- monocytes, CD16+ monocytes, cDCs and pDCs (**Supplementary Figure 2**). The distribution of cell types per donor is shown in **Supplementary Figure 3**. Single-cell RNA sequencing data can be found under GEO: GSE198891.

### 2.4 Data analysis and statistics

Flow cytometry data was analyzed with Kaluza v2.1 software (Beckman coulter, IN, USA) to obtain frequencies of positive cells and mean fluorescence intensities (MFI) of the cellular markers.

t-distributed Stochastic Neighbor Embedding (t-SNE) analyses were performed using FCS express version 6 (*De Novo* software, CA, USA). To this end, compensation was applied before subsequently gating APCs in all FCS files individually. APCs were gated as HLA-DR+CD19- single cells. Equal numbers of APCs were exported as separate files from each file and merged into a single FCS file including a file identifier. t-SNE was calculated based on expression of CD14, CD16, CD303, CD1c, CD141, PDL1, CD40, CD86, TLR2 and CD11c. Sampling options included an interval down sampling method, a Barnes- Hut approximation of 0.50, perplexity set to 60 and number of iterations to 2000.

Mann-Whitney U tests were performed to compare between GCA/PMR patients and HCs. Spearman rank correlations were calculated when indicated in the text. R (version 3.6.2) with the Seurat package (version 3.2.0) was used as described previously to analyze the scRNAseq data. All plots were created using GraphPad Prism version 9. P values <0.05 were considered statistically significant.

## 3 Results

### 3.1 t-SNE analyses reveal differential clustering of APC counts in GCA/PMR patients compared to HCs

**Figure 1:**
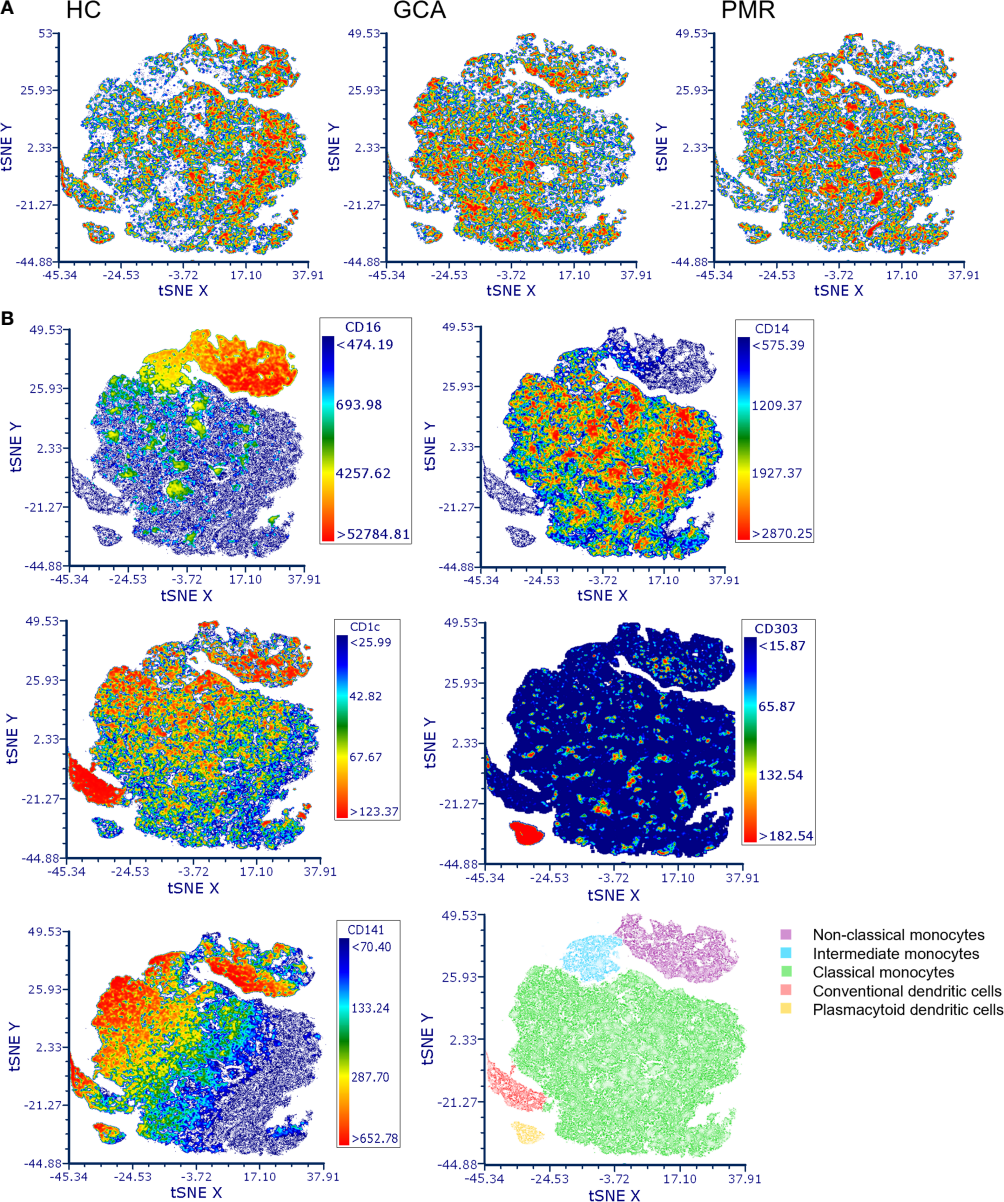
t-distributed Stochastic Neighbor Embedding (t-SNE) visualization of antigen presenting cell counts. **(A)** t-SNE plots of antigen presenting cell counts in HCs, GCA and PMR patients. **(B)** Marker expression of CD16, CD14, CD1c, CD141 and CD303 in the combined t-SNE plots of HCs and GCA/PMR patients for the identification of clusters with non-classical monocytes (CD14lowCD16+), intermediate monocytes (CD16+CD14+), classical monocytes (CD14+CD16-), conventional dendritic cells (CD141/CD1c+) and plasmacytoid dendritic cells (CD303+). HC, healthy control; GCA, giant cell arteritis; PMR, polymyalgia rheumatica; t-SNE, t-distributed Stochastic Neighbor Embedding.

t-SNE plots were made to assess the distribution of different APC subsets between HCs and patients with GCA and PMR (**Figure 1**). Clear distribution differences could be observed between HCs and GCA patients, and between HCs and PMR patients. t-SNE plots between GCA and PMR patients showed a similar distribution of counts. Visualization of the expression of CD16, CD14, CD1c, CD141 and CD303 in de t-SNE of total counts revealed five major subsets: non-classical monocytes (CD14lowCD16+), intermediate monocytes (CD16+CD14+), classical monocytes (CD14+CD16-), cDCs (CD141/CD1c+) and pDCs (CD303+). In the t-SNE plots of HCs and GCA/PMR patients we observed a shift in cell counts within the clusters of classical monocytes, non-classical monocytes, intermediate monocytes and cDCs but not in the pDCs.

### 3.2 Proportions and absolute counts of monocyte and DC subsets are affected in GCA and PMR

**Figure 2:**
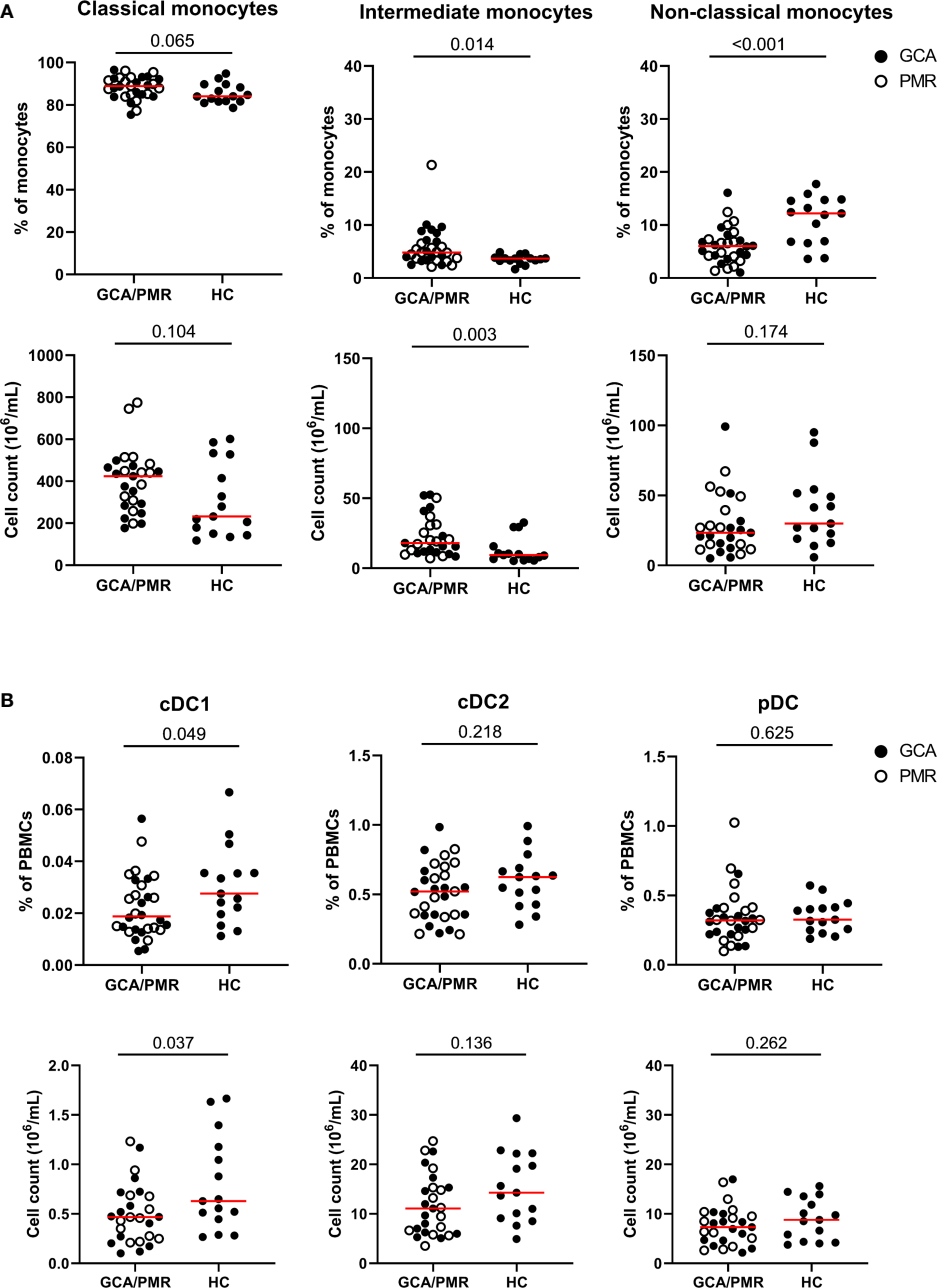
Frequencies and counts of monocytes and dendritic cell subsets. **(A)** Frequencies of classical monocytes, intermediate monocytes and non-classical monocytes among total monocytes (upper row) and cell counts (lower row) in GCA/PMR and HCs. **(B)** Frequencies of cDC1, cDC2 and pDCs among total PBMCs (upper row) and absolute cell counts (lower row) in GCA/PMR and HCs. Red line depicts the median. Closed circles represent GCA patients and open circles PMR patients. GCA, giant cell arteritis; PMR, polymyalgia rheumatica; HC, healthy controls; cDC1, conventional dendritic cell subset 1; cDC2, conventional dendritic cell subset 2; pDC, plasmacytoid dendritic cell. Statistical significance by Mann-Whitney U tests is indicated and p values are reported in the graphs.

Conventional gating strategies as shown in **Supplementary Figure 1** were used to calculate the frequencies of monocytes and DC subsets (**Figure 2**). GCA and PMR patients were pooled in the analysis, as t-SNE plots showed a similar distribution of subsets (**Figure 1**) and they have been postulated to be part of an overlapping syndrome (GPSD). Absolute counts of monocyte and DC subsets were calculated based on the frequencies and the absolute counts of total PBMCs (lymphocytes + monocytes). Higher proportions and absolute counts of intermediate monocytes were found in GCA/PMR patients than in HCs. Proportions of classical monocytes among total monocytes seemed to be slightly higher in GCA/PMR patients (trend, p=0.065) and absolute counts of classical monocytes appeared to show a similar pattern, albeit not statistically significant due to the large variation within the groups. In contrast, proportions of non-classical monocytes were reduced in GCA/PMR patients compared to HCs (**Figure 2A**). Analyses of DC subsets showed that proportions and absolute counts of the small population of cDC1 cells were reduced in GCA/PMR patients compared to HC whereas the cDC2 and pDC subset remained unchanged (**Figure 2B**). As a sub-analysis, we did compare whether patients with GCA had differences in monocyte and DC subset counts compared to patients with PMR, but no differences were found (**Supplementary Figure 4**).

Next, we assessed whether the proportions of circulating monocyte and DC subsets associated with systemic inflammation, as indicated by a high CRP (**Table 1**). The percentage of cDC1 (R=-0.41) and cDC2 (R=-0.52) within total PBMCs showed a moderately strong, negative, association with the CRP in GCA/PMR patients. This indicates that patients with a strong systemic inflammatory response have a relatively low proportion of circulating cDCs. Within the monocyte population, it appears that a shift from classical monocytes towards non-classical monocytes was also associated with a lower CRP.

**Table 1:** Associations of proportions of monocyte and dendritic cell (DC) subsets with C-reactive protein (CRP) levels at the time of GCA/PMR diagnosis, as a reflection of systemic inflammation.

	Spearman R correlation with CRP at diagnosis (mg/L)	
	As % of total PBMCs	As % of total monocytes
**Classical monocytes**	0.26	0.32 #
**Intermediate monocytes**	-0.01	-0.17
**Non-classical monocytes**	-0.20	-0.35 *
**cDC1**	-0.41 *	
**cDC2**	-0.52 *	
**pDC**	-0.24	

Shown are the Spearman R coefficients of the correlation between the percentage of each subset and the CRP. *: p<0.05, #: p<0.10 (trend). PBMCs, peripheral blood mononuclear cells.

### 3.3 TLR2 expression is changed in monocytes but not in DCs of patients with GCA/PMR

**Figure 3:**
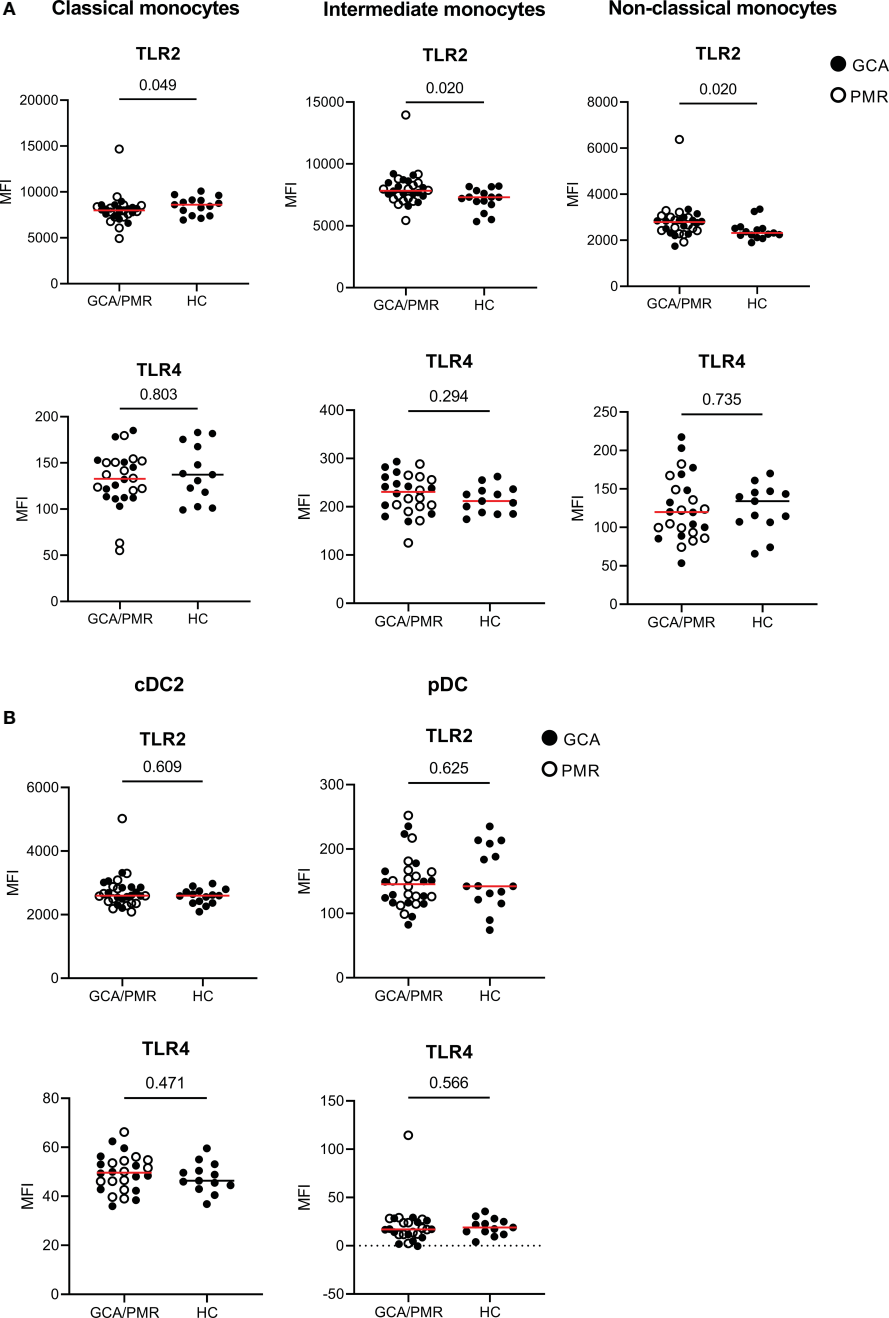
Expression of TLRs on APCs in GCA/PMR. Expression of TLR2 and TLR4 on monocyte subsets **(A)** and DC subsets **(B)**. Graphs illustrate the mean fluorescence intensity (MFI). Red line depicts the median. Closed circles represent GCA patients and open circles PMR patients. GCA, giant cell arteritis; PMR, polymyalgia rheumatica; HC, healthy controls; TLR, toll-like receptor. Statistical significance by Mann-Whitney U tests is indicated and p values are reported in the graphs.

To assess changes in expression of pattern and damage recognition receptors, we stained for TLR2 and TLR4 within monocytes and DC subsets (**Figure 3**). TLR2 expression appeared to be higher in intermediate and non-classical monocytes in GCA/PMR patients than in HCs. In contrast, classical monocytes expressed less TLR2 in GCA/PMR. TLR2 expression was comparable between patients and controls within DC subsets. In addition, TLR4 was not differently expressed either in both monocytes and DC subsets.

### 3.4 Classical monocytes show signs of reduced activation in GPSD; non-classical monocytes show higher expression of activation markers

**Figure 4:**
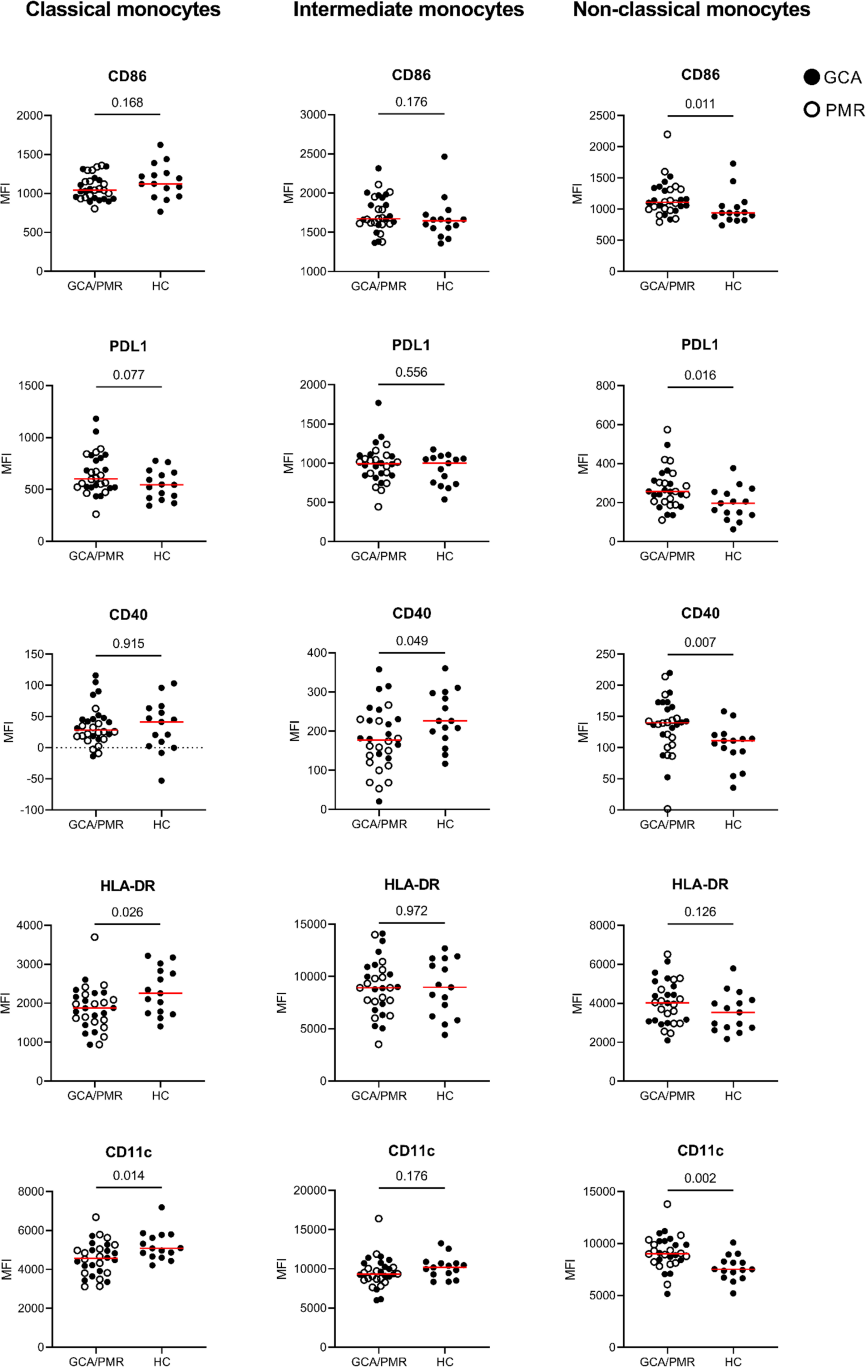
Mean fluorescence intensity of APC-associated markers on monocytes. Graphs illustrate the mean fluorescence intensity (MFI) of CD86, PDL1, CD40, HLA-DR and CD11c on classical monocytes, intermediate monocytes and non-classical monocytes in GCA/PMR and HCs. Red line depicts the median. Closed circles represent GCA patients and open circles PMR patients. GCA, giant cell arteritis; PMR, polymyalgia rheumatica; HC, healthy controls; PDL1, programmed death ligand 1; HLA-DR, human leukocyte antigen-DR. Statistical significance by Mann-Whitney U tests is indicated and p values are reported in the graphs.

After assessing the expression of TLRs we investigated whether the expression of the immune checkpoints CD86, PDL1 and CD40 and the activation markers HLA-DR and CD11c was different in GCA/PMR as well. To this end we determined and compared the mean fluorescence intensity of these markers in each monocyte subset between GCA/PMR patients and HC (**Figure 4**).

Interestingly, whereas classical monocytes show a reduced expression of HLA-DR and CD11c which could be indicative of a reduced activation, non-classical monocytes show an opposite pattern in GCA/PMR. Non-classical monocytes in GCA/PMR had elevated per cell expression of CD11c, but also of CD86 and CD40. PDL1 appeared to be elevated in non-classical monocytes and showed a trend towards higher expression in classical monocytes in GCA/PMR. Expression of HLA-DR on non-classical monocytes remained unchanged between the study groups. Intermediate monocytes of GCA/PMR patients had, in contrast to non-classical monocytes, a reduced expression of CD40 and showed no differences in the expression of other markers. When comparing patients with GCA and patients with PMR, we found that CD40 expression on classical and intermediate monocytes was particularly low in PMR (p<0.05, **Supplementary Figure 5**). No other differences were found in expression levels between patients with GCA and PMR.

In summary, classical monocytes of GCA/PMR patients overall appeared to be less activated, whereas non-classical monocytes showed a higher expression of activation markers compared to HCs.

### 3.5 Reduced expression of activation markers in cDC2 and aberrant expression of immune checkpoints in pDCs in GCA/PMR

**Figure 5:**
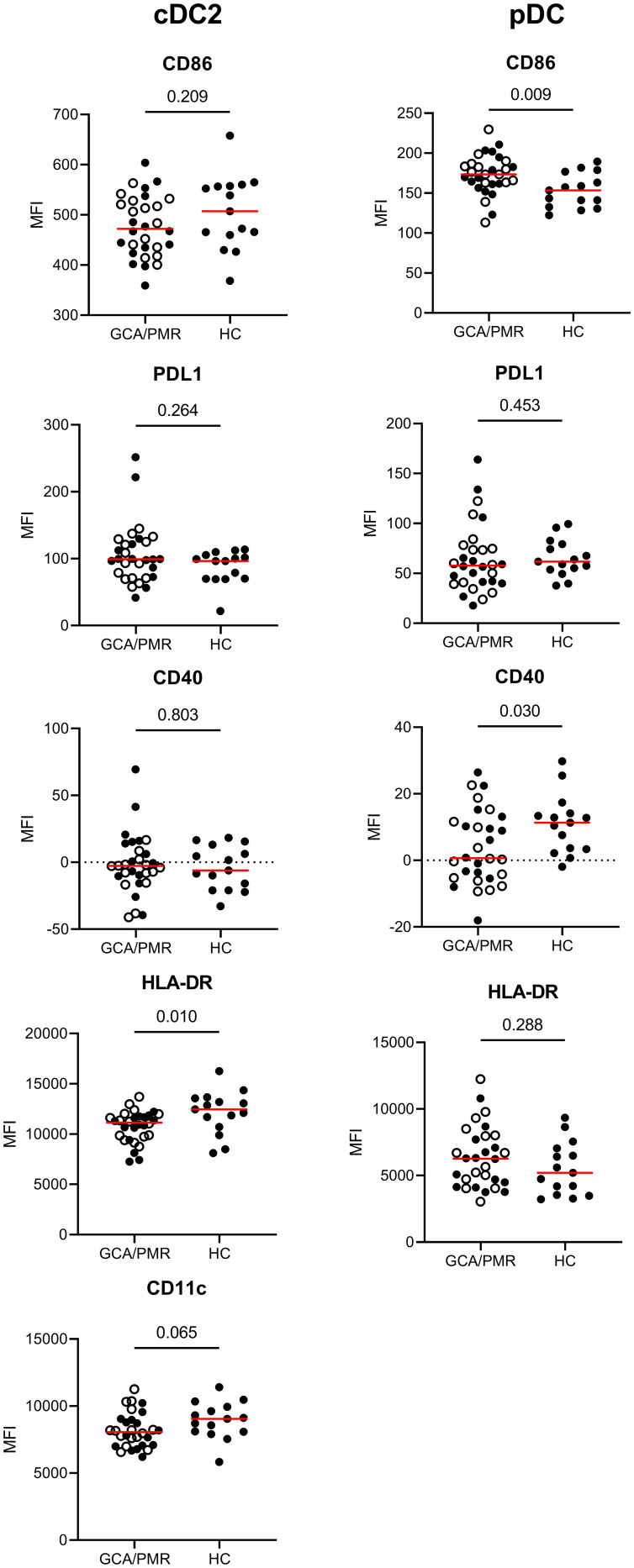
Mean fluorescence intensity of APC-associated markers on dendritic cells. Graphs illustrate the mean fluorescence intensity (MFI) of CD86, PDL1, CD40, HLA-DR and CD11c on cDC2s and pDCs in GCA/PMR and HCs. Red line depicts the median. Closed circles represent GCA patients and open circles PMR patients. cDC2, conventional dendritic cell 2; pDC, plasmacytoid dendritic cell; GCA, giant cell arteritis; PMR, polymyalgia rheumatica; HC, healthy controls; PDL1, programmed death ligand 1; HLA-DR, human leukocyte antigen-DR. Statistical significance by Mann-Whitney U tests is indicated and p values are reported in the graphs.

After assessing the surface marker expression by monocytes, we assessed the same surface marker expression by cDC2s and pDCs (**Figure 5**). As the population of cDC1 cells was very small, expression of the surface markers by cDC1 cells could not be determined. cDC2 and pDCs had different expression levels between GCA/PMR and HCs. cDC2 had a reduced expression of HLA-DR and CD11c (trend, p=0,065) compared to HCs whereas these markers were unchanged within the pDC subset. pDCs however, had higher expression of CD86 in GCA/PMR patients and lower expression of CD40. No changes were found regarding PDL1 expression in DC subsets of patients and controls.

### 3.6 Single-cell RNA sequencing of monocytes and DCs in GCA

**Figure 6:**
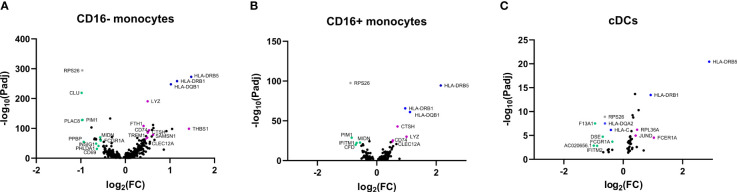
Volcano plots of differentially expressed genes in APCs from GCA patients and HCs. Differentially expressed genes in **(A)** CD16- monocytes, **(B)** CD16+ monocytes, **(C)** cDCs between HCs and GCA patients (n=3). Purple dots show highlighted genes that are downregulated in GCA patients, green dots are genes upregulated in GCA patients, grey dots are genes that are differentially expressed but show high donor specificity and blue dots are genes differentially expressed due to HLA type differences between donors. Only statistically significant differentially expressed genes are indicated in the graphs.

To gain more insight into the function of APCs and potential areas of future research in monocytes and DCs, we utilized the previously published scRNAseq dataset obtained by our group performed in PBMCs of three GCA patients and three HCs (18). In **Figure 6** three volcano plots are shown that illustrate differentially expressed genes within CD16- monocytes, CD16+ monocytes and cDCs. Genes of interest have been highlighted in the graphs in green and purple. Some genes (in blue) have a high fold change between HC and GCA but are related to HLA type. Genes in grey are previously shown to be highly donor specific (e.g. *RPS26*) (18). The complete list of genes with a fold change of >0,4 and an adjusted p value of <0.05 can be found in the **Supplementary**. Results in pDCs are not shown, as only two genes were differentially expressed: *RPS26*, which was donor specific, and *HLA-DRB5*, related to HLA type.

Some genes that are higher expressed by GCA patients than HCs, are related to immune function and inflammation. Enhanced gene expression by CD16- monocytes in GCA patients included for instance the expression of *CLU*, which is related to a protein involved in TNF-α secretion by macrophages (19) and phagocytosis of late apoptotic cells (20). Other genes that had a higher expression in GCA were *PLAC8*, previously found to upregulated on activated monocytes (21), *PIM1* which is related to a protein regulating proinflammatory cytokine responses in synoviocytes (22) and *PHLDA1*, which was recently found to be related to TLR4 activity (23). In addition, increased expression of *PPBP*, encoding CXCL7 was found in CD16- monocytes of GCA patients as well. CD16+ monocytes showed upregulation of *PIM1* as well, and in addition upregulation of *CFD*, related to the complement system and *IFITM1*, related to the interferon (IFN) pathway. Other genes, such as *INSIG1* and *MIDN*, which were upregulated on monocytes are associated with metabolism and regulators of glucokinase activity.

Decreased expression of genes in GCA patients included *THBS1* in CD16- monocytes, encoding thrombospondin-1. Thrombospondin-1 has been associated with anti-inflammatory properties by being secreted by apoptotic monocytes, to mediate engulfment and decrease immune responses (24). Mice deficient for thrombospondin-1 have defective IL-10 production by macrophages and deficient inflammation resolution after injury (25). Despite exerting anti-inflammatory functions, thrombospondin-1 is associated with vascular inflammation properties as well, as evidenced by a *THBS1* knockout mouse model of chemically induced abdominal aortic aneurysm (26). *TREM1* was downregulated as well and encodes for proteins associated with monocyte activation after acute bacterial and fungal infections (27). Interestingly, both monocyte subsets had decreased expression of *CLEC12A* in GCA, encoding for proteins that negatively regulate inflammation (28).

Many of the genes that were differentially expressed in cDCs of GCA patients have an undefined function in DCs. Of interest is the upregulation of the *IFITM2* gene, which is like *IFITM1* related to the IFN pathway. Furthermore, *FCER1A*, related to DC regulation was downregulated in GCA (29).

Together, these results indicate that GCA patients had differentially expressed genes within monocytes and DCs related to inflammation and metabolism.

## 4 Discussion

Our study provides new insights into the role of monocytes and DCs in the pathogenesis of GCA and PMR. We found that patients with GCA/PMR exhibit a shift in monocyte subset proportions, with higher proportions of classical and intermediate monocytes, and reduced proportions of non-classical monocytes. Moreover, we observed a phenotypic shift in monocytes of GCA/PMR patients. Classical monocytes showed reduced expression of TLR2, HLA-DR, and CD11c, whereas non-classical monocytes exhibited higher expression of TLR2, CD86, CD40, and CD11c. Furthermore, both classical (trend) and non-classical monocytes had a higher expression of PDL1. Additionally, our results indicate that GCA/PMR patients exhibit lower percentages of the cDC1 subset and reduced expression of HLA-DR and CD11c (trend) on cDC2. Interestingly, CRP appears to be associated with a shift from non-classical monocytes to classical monocytes, as well as reduced percentages of cDC1 and cDC2 cells. These findings add to the knowledge on the pathogenesis of GCA/PMR and may have implications for the development of new therapeutic approaches.

The changes in the distribution of monocyte and DC subsets align with previous findings of our group and others in GCA/PMR patients and other inflammatory diseases. Reduced non-classical monocyte proportions have been described in our cohort before, in non-overlapping patients and controls (30). Circulating DC subsets have been studied less frequently in inflammatory diseases. Previously, lower counts of circulating cDCs have also been described in inflammatory conditions such as Sjögren’s syndrome (31), and other types of vascular inflammation, such as coronary artery disease (32). In this study we did not observe significantly reduced proportions of the main cDC subset, cDC2, however we did show that a lower proportion of these cells associates with a stronger systemic inflammation. This potentially reflects enhanced migration from the blood to the tissues in patients with a strong systemic inflammatory response. In patients with early rheumatoid arthritis, disease activity was also found to be associated with reduced proportions of cDCs (33). We did measure lower proportions of the rare CD141+ cDC1 subset, which also aligns with the aforementioned study in rheumatoid arthritis. That study however also reported evidence of enhanced activation (CD86 expression) of the cDC2 subset, which was not substantiated in our study, as we rather showed reduced HLA-DR expression on cDC2. Finally, the increased classical/intermediate proportions are not in line with the lack of expansion of non-classical monocyte and cDC1 counts, which indicates that there are potential defects in the developmental transitions from classical/intermediate monocytes towards these phenotypes.

The contrasting activation patterns observed in monocyte subsets of GCA/PMR patients; reduced expression in classical and enhanced activation of non-classical monocytes, has not been described before and could have several explanations. The exact functional similarities and differences between monocyte subsets has not been fully crystallized and therefore, a designation of pro- or anti-inflammatory subsets is inappropriate. Non-classical monocytes, and to a larger extent intermediate monocytes, are known for their heightened ability to present antigens and sense PAMPs and DAMPs. This has been reported before and is supported by higher expression levels of markers such as CD86, HLA-DR and TLRs, compared to classical monocytes (34). However, classical monocytes are more equipped to produce high amounts of pro-inflammatory cytokines (9). Monocytes likely follow a linear differentiation pattern from classical to intermediate to non-classical monocytes. The differentiation step from intermediate to non-classical monocytes could occur outside the blood (35). The increased activation marker expression by non-classical monocytes could indicate increased antigen expression activity and involvement in the disease pathogenesis. Also, in other (auto)inflammatory diseases such as systemic lupus erythematosus (SLE) and rheumatoid arthritis (RA) involvement of non-classical monocytes in the development of the disease has been reported (36). In SLE, non-classical monocytes seem to contribute to activation of T cells and B cells and (indirectly) to auto-antibody production. Furthermore, in a murine RA model, non-classical monocytes were able to differentiate into inflammatory macrophages.

The decreased activation marker expression on classical monocytes in GCA/PMR is less expected, due to their capacity to produce high amounts of pro-inflammatory cytokines. Their reduced expression could indicate a state of exhaustion caused by either long-term inflammation or long-term exposure to viruses (37–39). This is also supported by the trend towards increased expression of PDL1. However, this explanation is less likely as this would be expected to affect the intermediate and non-classical monocytes as well. The finding that non-classical monocytes show higher activation markers alongside increased expression of PDL1 in GCA/PMR makes this explanation even less likely. Indeed, PDL1 expression, just as PD1, has been known to be upregulated in response to pro-inflammatory stimuli, as a counter mechanism to dampen the inflammation (reviewed in (40)).

As monocytes have a high turn-over rate, reduced expression of activation markers in classical monocytes of GCA/PMR papers could also be indicative of a large pool of recently recruited classical monocytes from the bone marrow (35). Recent studies on patients with mild COVID-19 or myocardial infarction demonstrated changes to monocyte subsets equivalent to the findings in this study: a disappearance of non-classical monocytes and an accumulation of proinflammatory classical monocytes with low HLA-DR expression (41, 42). The authors linked these changes to high serum levels of IL-6 and calprotectin (S100A8/S100A9), which our group also reported on in GCA/PMR (43), and potentially contributes to accelerated trans-endothelial migration of monocytes.

In addition to our flow cytometry analysis, we also employed an scRNA sequencing pilot study on APCs in GCA patients and HCs, in which we detected mainly aberrations in monocytes and cDCs, rather than pDCs. In general, these findings point at an upregulation of genes involved in glucose metabolism and proinflammatory responses, and a downregulation of genes involved in the regulation of immune response. Our group have previously reported on metabolically active APCs in GCA patients which is in line with these findings (44). Taken together, these findings hint at a shifted balance in APCs of GCA patients toward a dysregulated, proinflammatory phenotype of APCs in GCA patients. The genes identified could be targets to study in larger scale studies, that should also validate whether these changes translate to the protein level.

The present study has several strengths that contribute to our understanding of the role of monocytes and DCs in GCA and PMR. Firstly, we focused on newly-diagnosed, well-characterized patients, and age-matched controls, which reduces the potential confounding effects of treatment and other factors. Secondly, we conducted in-depth phenotyping of monocytes and DCs, including the measurement of absolute counts, and employed sophisticated analysis methods such as tSNEs. Finally, we included an analysis of scRNA sequencing data which could provide further clues on disease-specific processes. However, there are also some weaknesses that should be considered. Firstly, the sample size is relatively small, which limits the statistical power and generalizability of our findings. Secondly, we did not include any functional data, such as the ability of monocytes and DCs to respond to stimuli or their ability to induce T cell activation. For pDCs, other, more relevant TLRs such as TLR7 and TLR9 should be studied in future studies as well. Also, the number of patients and controls was particularly limited for the scRNA sequencing data, and only patients with GCA, not PMR were included. Lastly, it is challenging to understand how the changes in circulating monocytes and DCs that we observed relate to the tissue-specific processes that occur in the inflamed arteries and synovia of GCA and PMR patients.

In conclusion, our study provides novel insights on monocytes and DCs in GCA and PMR patients. We observed a shift in monocyte subset proportions and phenotypic changes in monocytes of GCA/PMR patients, as well as altered percentages of DC subsets. However, the question of how these phenotypic changes in blood cells associate with the processes occurring at the inflammatory sites remains unanswered. Nonetheless, our study provides important insights into the systemic immune changes that occur in GCA and PMR and lays the groundwork for future studies to address these limitations. Potentially, these studies could explore the potential therapeutic implications of targeting monocytes and DCs in GCA/PMR.

## Data availability statement

Flow cytometry datasets are available on request: The raw data supporting the conclusions of this article will be made available by the authors, without undue reservation. Single-cell RNA sequencing data can be found under GEO: GSE198891.

## Ethics statement

The studies involving human participants were reviewed and approved by the UMCG METc, METc2010/222. The patients/participants provided their written informed consent to participate in this study.

## Author contributions

YS, RR, EB, KG, WA, and PH contributed to conception and design of the study. B-CH, YS, and RR performed the experiments. YS and RR performed the statistical analysis. All authors contributed to the article and approved the submitted version.
