# Supplementary material for: Aberrant phenotype of circulating antigen presenting cells in giant cell arteritis and polymyalgia rheumatica
Source: Front Immunol. 2023 Aug 2;14:1201575. doi: 10.3389/fimmu.2023.1201575 (PMC10433739; doi:10.3389/fimmu.2023.1201575)
Supplement: Supplementary file 1 [file DataSheet_1.zip › Supplementary files.docx]

Supplementary Material

Aberrant Phenotype of Circulating Antigen Presenting Cells in Giant Cell Arteritis and Polymyalgia Rheumatica

Rosanne D. Reitsema^1,2^, Bernd-Cornèl Hesselink^1^, Wayel H. Abdulahad^1,3^, Kornelis S.M. van der Geest^1^, Elisabeth Brouwer^1^, Peter Heeringa^3^, Yannick van Sleen^1^

^1^ Department of Rheumatology and Clinical Immunology, University of Groningen, University Medical Center Groningen, Groningen, The Netherlands.

^2^ School of Medical Sciences, Faculty of Medicine and Health, Örebro University, Örebro, Sweden.

^3^ Department of Pathology and Medical Biology, University of Groningen, University Medical Center Groningen, Groningen, The Netherlands.

*** Correspondence:**Yannick van Sleen
[y.van.sleen@umcg.nl](mailto:y.van.sleen@umcg.nl)

# Supplementary Tables

## Supplementary Table 1: Study population information

|  | Age | CRP mg/L | | ESR mm/hr | | Hb mmol/L | TAB | PET GCA | PET PMR | PMR* | ACR EULAR for GCA 2022 | ACR EULAR for PMR 2012 |
| --- | --- | --- | --- | --- | --- | --- | --- | --- | --- | --- | --- | --- |
|  |  | |  | |  |  |  |  |  |  |  |  |
| GCA1 | 81 | 11 | | 23 | | 9,2 | + | NA | NA | - | Y |  |
| GCA2 | 79 | 5 | | 9 | | 8 | + | NA | NA | - | Y |  |
| GCA3 | 79 | 92 | | 81 | | 7,9 | + | NA | NA | - | Y |  |
| GCA4 | 59 | 122 | | 107 | | 6,6 | NA | + | - | - | N |  |
| GCA5 | 73 | 40 | | 64 | | 6,6 | + | - | - | - | Y |  |
| GCA6 | 67 | 76 | | 116 | | 5,9 | NA | + | + | + | Y | Y |
| GCA7 | 74 | 116 | | 94 | | 8,3 | - | - | - | - | Y |  |
| GCA8 | 64 | 63 | | 73 | | 6,1 | NA | NA | NA | - | Y |  |
| GCA9 | 71 | 42 | | 106 | | 6,5 | NA | + | + | + | Y | Y |
| GCA10 | 61 | 14 | | 31 | | 8,1 | NA | + | + | + | Y | N |
| GCA11 | 66 | 105 | | 99 | | 6,4 | NA | + | + | + | Y | Y |
| GCA12 | 92 | 64 | | 81 | | 8,2 | NA | NA | NA | - | Y |  |
| GCA13 | 58 | 29 | | 66 | | 7,9 | NA | + | + | + | Y | N |
| GCA14 | 54 | 17 | | 45 | | 7,9 | - | - | - | - | Y |  |
| GCA15 | 66 | 62 | | 38 | | 8,5 | NA | NA | NA | + | Y |  |
| PMR1 | 68 | 12 | | 24 | | 8,3 |  |  | + |  |  | Y |
| PMR2 | 71 | 29 | | 44 | | 8,5 |  |  | + |  |  | Y |
| PMR3 | 79 | 118 | | 89 | | 7,2 |  |  | NA |  |  | Y |
| PMR4 | 64 | 14 | | 72 | | 7,3 |  |  | + |  |  | Y |
| PMR5 | 82 | 68 | | 95 | | 8,3 |  |  | + |  |  | Y |
| PMR6 | 62 | 29 | | 67 | | 7,8 |  |  | NA |  |  | Y |
| PMR7 | 56 | 61 | | 72 | | 8,4 |  |  | + |  |  | Y |
| PMR8 | 52 | 53 | | 59 | | 7,1 |  |  | + |  |  | Y |
| PMR9 | 82 | 49 | | 62 | | 7,1 |  |  | + |  |  | Y |
| PMR10 | 68 | 12 | | 39 | | 7,6 |  |  | + |  |  | Y |
| PMR11 | 63 | 52 | | 79 | | 8,9 |  |  | + |  |  | Y |
| PMR12 | 70 | 0,6 | | 11 | | 9 |  |  | + |  |  | N |
| PMR13 | 68 | 37 | | 53 | | 8,3 |  |  | + |  |  | Y |
| PMR14 | 54 | 16 | | 28 | | 8,7 |  |  | + |  |  | Y |
| PMR15 | 69 | 35 | | 87 | | 7,6 |  |  | + |  |  | Y |
| HC1 | 67 | 5 | | 24 | | 7,6 |  |  |  |  |  |  |
| HC2 | 70 | 5 | | 28 | | 9,4 |  |  |  |  |  |  |
| HC3 | 73 | 5 | | 16 | | 9,2 |  |  |  |  |  |  |
| HC4 | 66 | 5 | | 8 | | 7,9 |  |  |  |  |  |  |
| HC5 | 73 | 5 | | 10 | | 8,1 |  |  |  |  |  |  |
| HC6 | 67 | 5 | | 9 | | 9 |  |  |  |  |  |  |
| HC7 | 75 | 5 | | 3 | | 10,2 |  |  |  |  |  |  |
| HC8 | 69 | 4,7 | | 26 | | 8,5 |  |  |  |  |  |  |
| HC9 | 68 | 2,4 | | 19 | | 8,4 |  |  |  |  |  |  |
| HC10 | 73 | 4 | | 5 | | 9 |  |  |  |  |  |  |
| HC11 | 70 | 5 | | 5 | | 8,5 |  |  |  |  |  |  |
| HC12 | 70 | 5 | | 6 | | 9,4 |  |  |  |  |  |  |
| HC13 | 60 | 5 | | 13 | | 9,1 |  |  |  |  |  |  |
| HC14 | 66 | 4 | | 10 | | 9,6 |  |  |  |  |  |  |
| HC15 | 58 | 5 | | 3 | | 8,3 |  |  |  |  |  |  |

*GCA patients diagnosed with PMR as well.
TAB: temporal artery biopsy, PET: [^18^F]fluorodeoxyglucose-Positron Emission Tomography-scan, NA: not applicable or not determined.

## Supplementary Table 2: Antibodies used for flow cytometry

| **Marker** | **Fluorochrome** | **Company** | **Catalogue number** |
| --- | --- | --- | --- |
| CD1c | BUV395 | BD Biosciences | 742751 |
| CD11c | APC | BD Biosciences | 333144 |
| CD14 | Pacific Orange | Life Technologies | MHCD1430 |
| CD16 | BUV737 | BD Biosciences | 564434 |
| CD19 | AF-700 | Thermo Fisher Scientific | 56-0199-42 |
| CD40 | APC-Cy | BioLegend | 334224 |
| CD86 | BB515 | BD Biosciences | 564544 |
| CD141 | BB700 | BD Biosciences | 742245 |
| CD303 | BV785 | BioLegend | 354222 |
| HLA-DR | V450 | BD Biosciences | 655874 |
| PD-L1 | PE-Cy7 | BioLegend | 329715 |
| TLR2 | BV650 | BD Biosciences | 742769 |
| TLR4 | BV711 | BD Biosciences | 564404 |

# Supplementary Figures

## Supplementary Figure 1

**A**


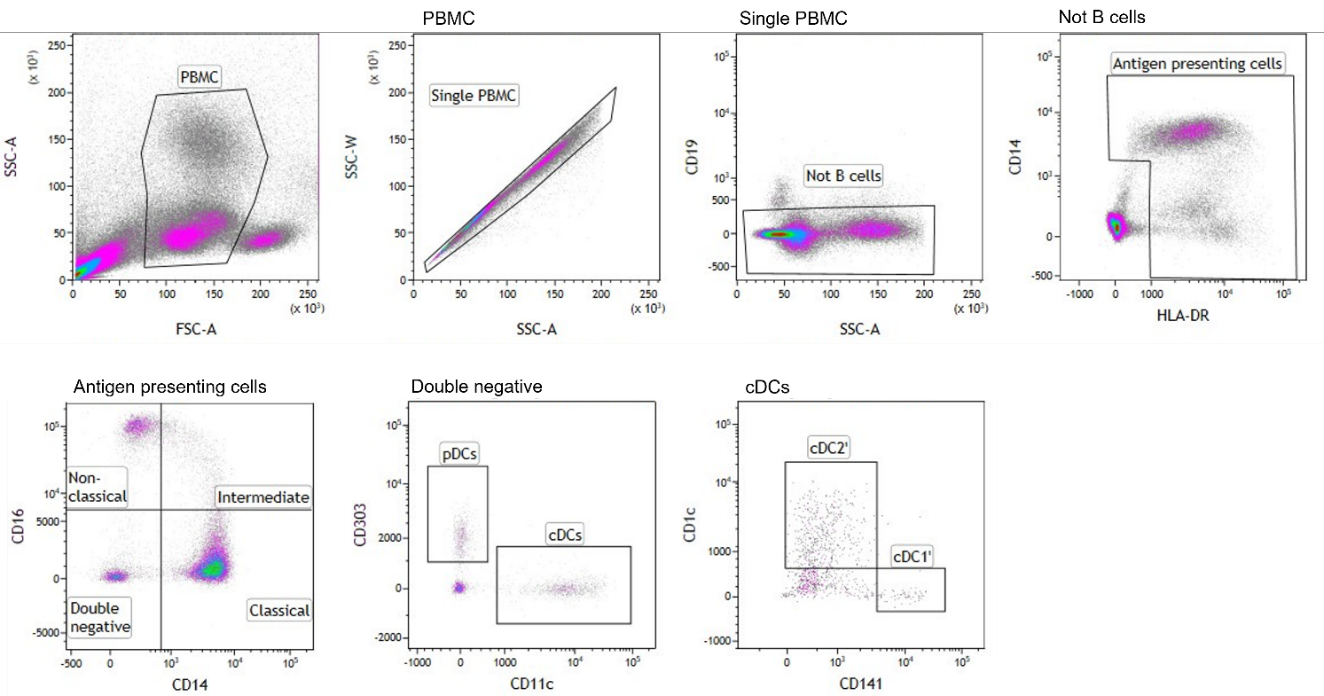

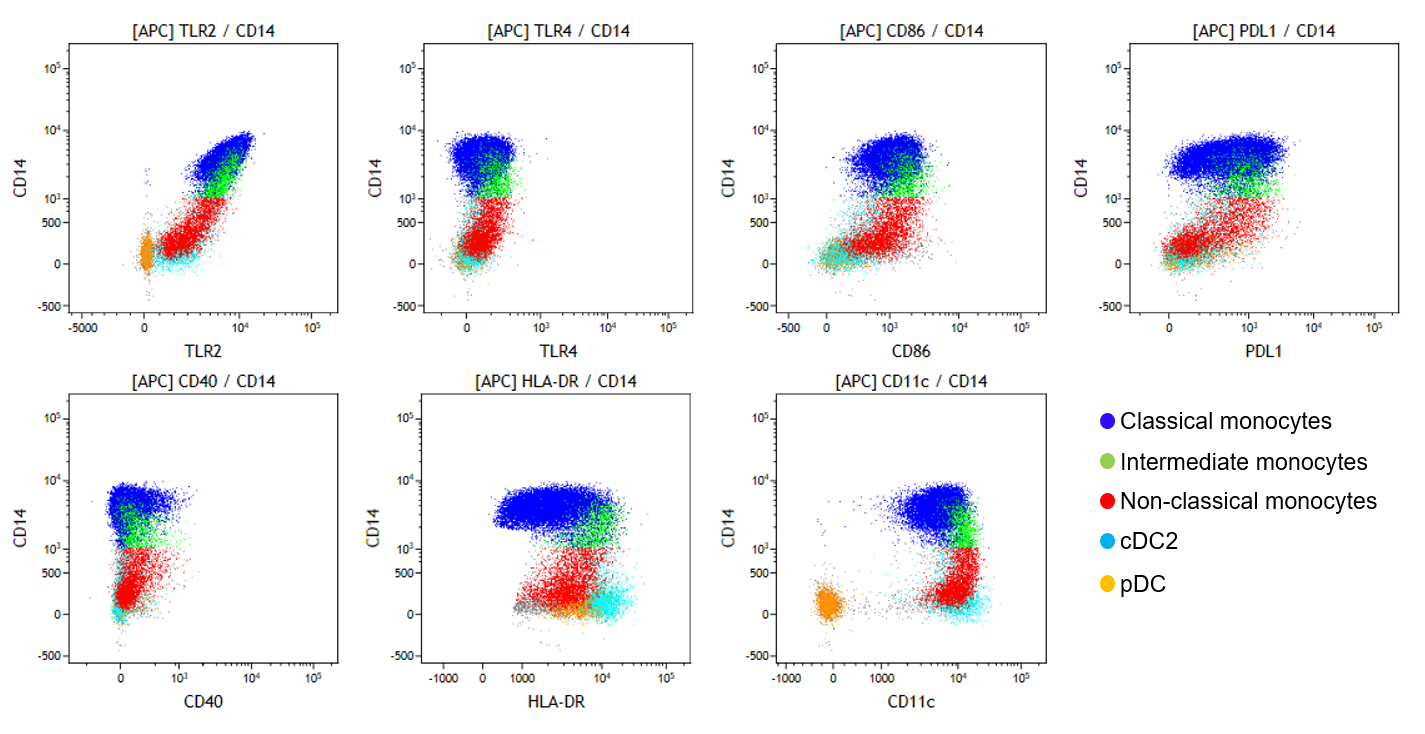
**Supplementary Figure 1: Gating strategy and marker expression in the flow cytometry experiments**. A: Single PBMCs were selected based on size and granularity. CD19 was used to gate out B cells. CD14 and HLA-DR was used to select all antigen presenting cells. Antigen presenting cells were further classified into non-classical, intermediate and classical monocytes based on CD16 and CD14 expression. CD16/CD14 double negative cells were divided into pDC based on CD303 and cDCs based on CD11c expression. cDC2 cells were CD1c positive and cDC1 cells were CD141 positive. B: Shown is the expression of each of the markers of interest against CD14 expression for the total antigen-presenting cell (APC) population of a representative sample.

**B**

## Supplementary Figure 2


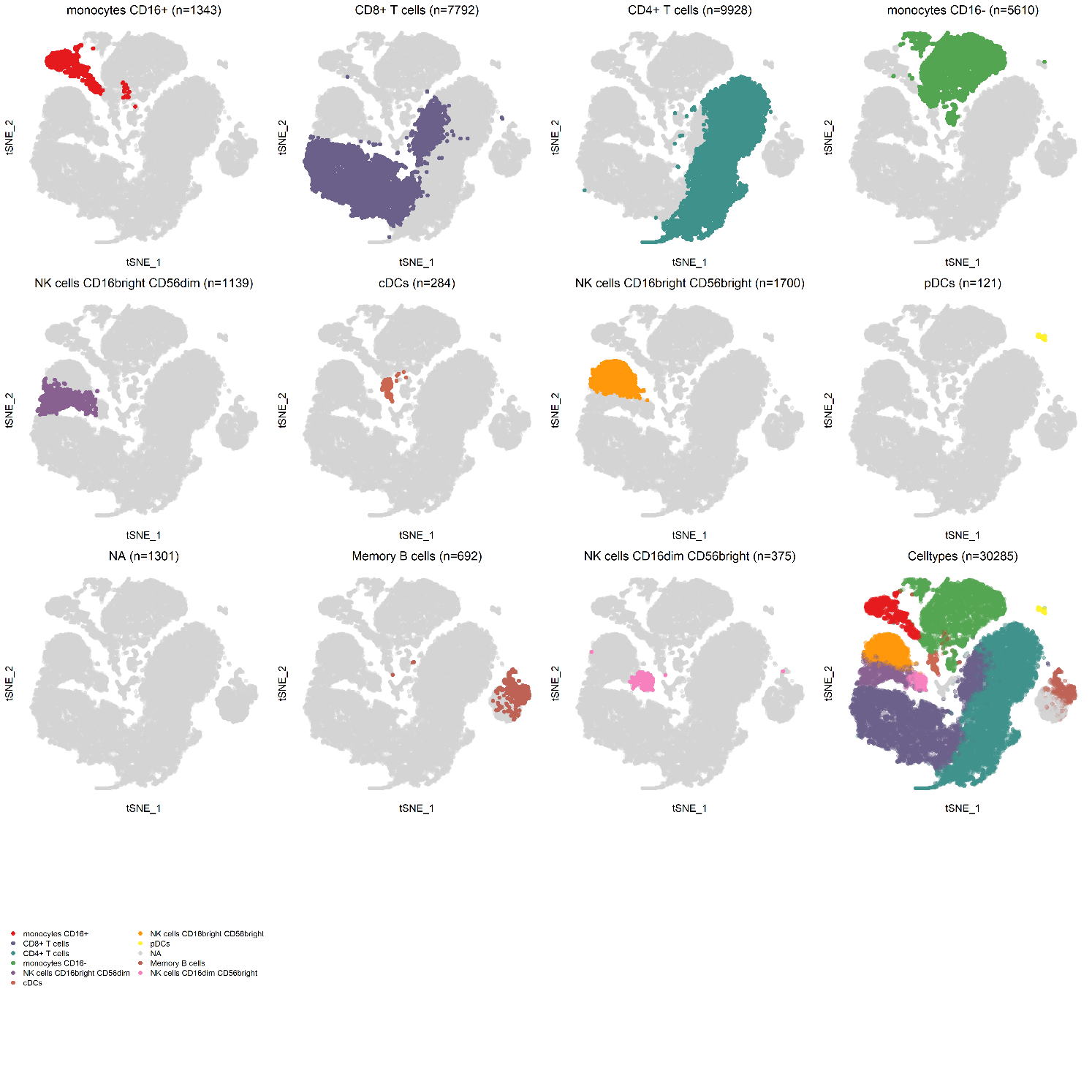


**Supplementary Figure 2: t-SNE representation of all immune cell subsets in GCA and HCs.** In the single-cell RNA sequencing dataset immune cell types were assigned to clusters. t-SNE plots show the distribution of immune cell types, including CD16- and CD16+ monocytes, cDCs and pDCs in GCA patients and HCs (n=3). CD16+ and CD16- monocytes were identified as *CD14-FCGR3A+* and *CD14+FCGR3A-*, respectively. pDCs had high expression of *CLEC4C* and cDCs expressed *CD1c* or *CD141*.

## Supplementary Figure 3


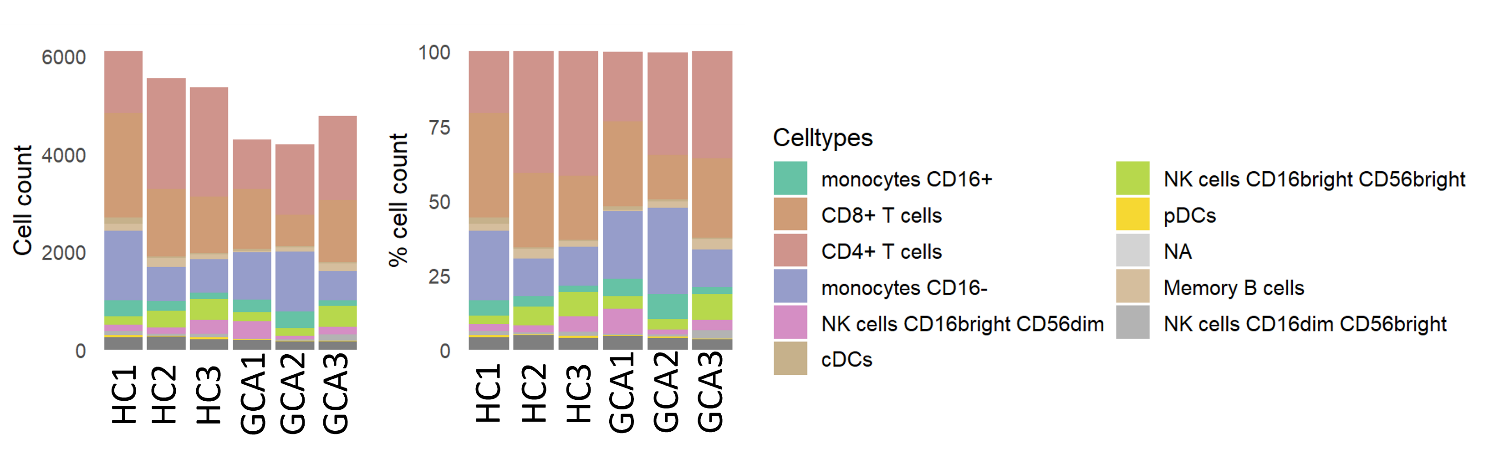


**Supplementary Figure 3: Distribution of cell types per donor in the scRNAseq dataset.** Distribution of identified cell subsets in the scRNAseq data per donor. Both the absolute numbers as the percentage of total immune cells are expressed. HC: healthy control, GCA: giant cell arteritis.

## Supplementary Figure 4


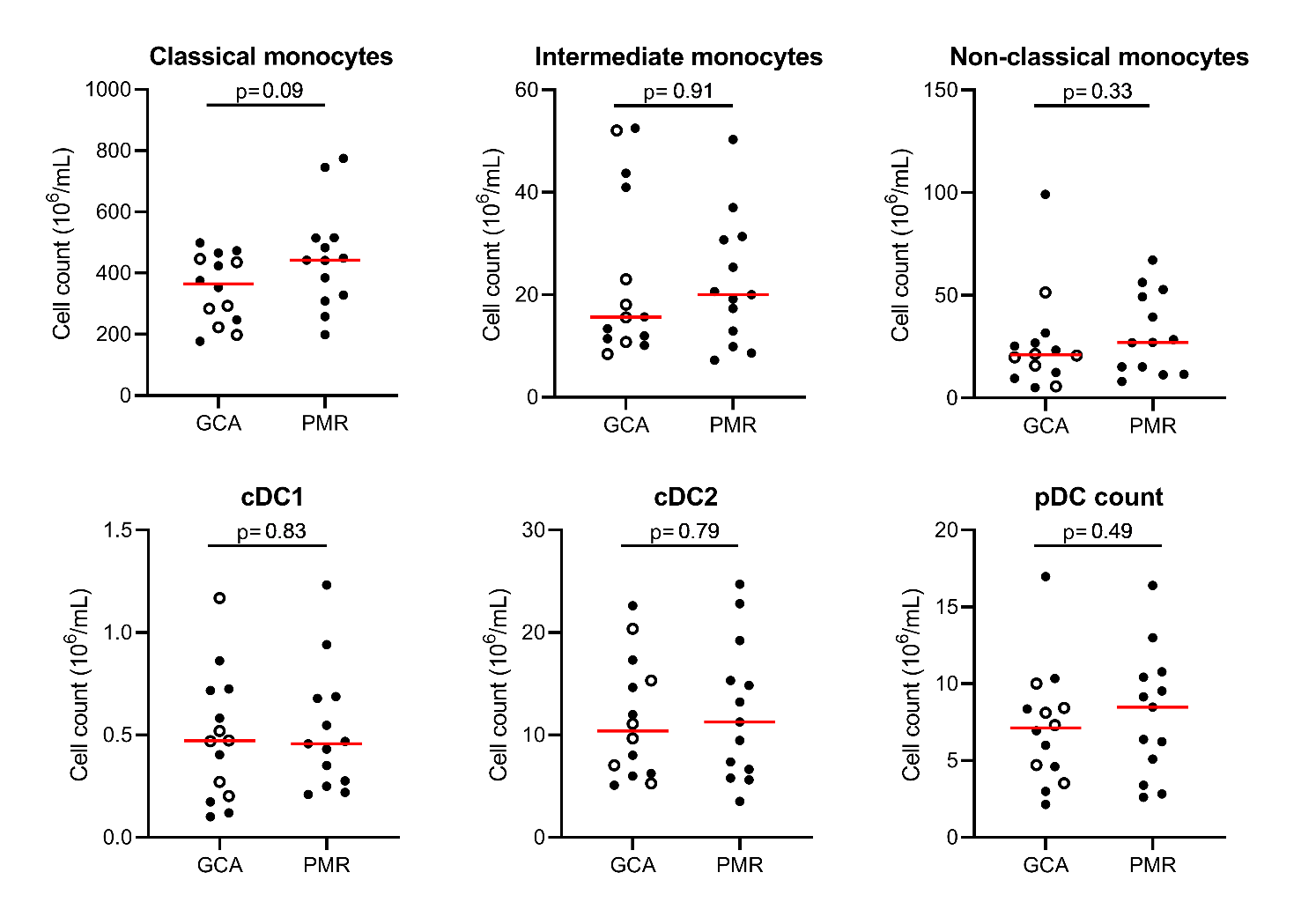


**Supplementary Figure 4: Cell counts of monocytes and dendritic cell subsets for GCA versus PMR patients.** Open circles in the GCA group represent GCA patients that presented with overlapping PMR symptoms at diagnosis. Statistical significance by Mann-Whitney U tests is indicated and p values are reported in the graphs.

## Supplementary figure 5

**
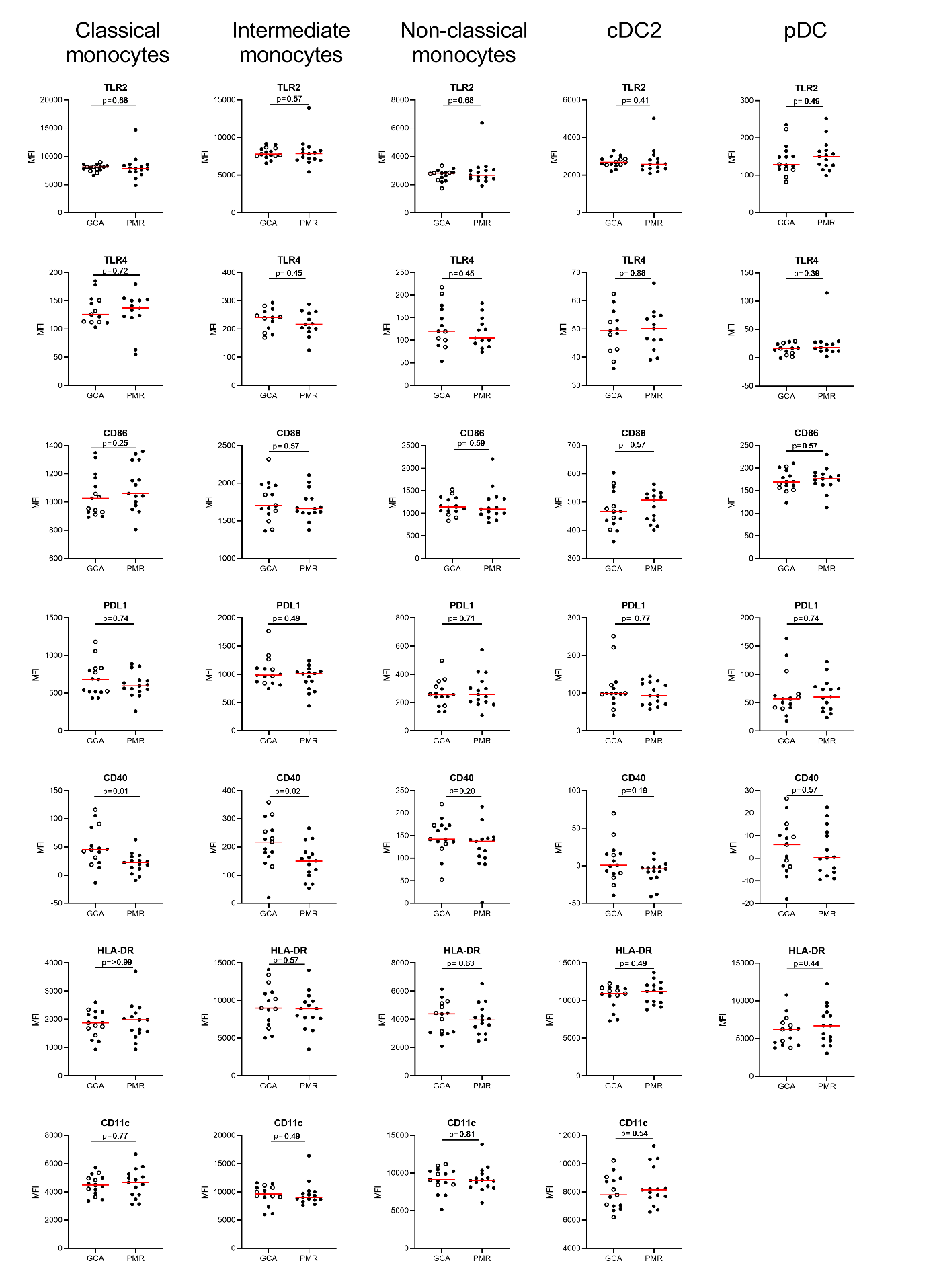

Supplementary Figure 5: Expression of TLRs and activation markers on monocyte and DC subsets for GCA versus PMR patients.** Open circles in the GCA group represent GCA patients that presented with overlapping PMR symptoms at diagnosis. Statistical significance by Mann-Whitney U tests is indicated and p values are reported in the graphs.
